# Horses Categorize Human Emotions Cross-Modally Based on Facial Expression and Non-Verbal Vocalizations

**DOI:** 10.3390/ani9110862

**Published:** 2019-10-24

**Authors:** Miléna Trösch, Florent Cuzol, Céline Parias, Ludovic Calandreau, Raymond Nowak, Léa Lansade

**Affiliations:** INRA, PRC, CNRS, IFCE, Université de Tours, 37380 Nouzilly, France; florent.cuzol@gmail.com (F.C.); celine.parias@inra.fr (C.P.); ludovic.calandreau@inra.fr (L.C.); raymond.nowak@inra.fr (R.N.); lea.lansade@inra.fr (L.L.)

**Keywords:** *Equus caballus*, social cognition, human-animal relationship, emotions

## Abstract

**Simple Summary:**

Recently, an increasing number of studies have investigated the expression and perception of emotions by non-human animals. In particular, it is of interest to determine whether animals can link emotion stimuli of different modalities (e.g., visual and oral) based on the emotions that are expressed (i.e., to recognize emotions cross-modally). For domestic species that share a close relationship with humans, we might even wonder whether this ability extends to human emotions. Here, we investigated whether domestic horses recognize human emotions cross-modally. We simultaneously presented two animated pictures of human facial expressions, one typical of joy and the other of anger; simultaneously, a speaker played a human non-verbal vocalization expressing joy or anger. Horses looked at the picture that did not match the emotion of the vocalization more (probably because they were intrigued by the paradoxical combination). Moreover, their behavior and heart rate differed depending on the vocalization: they reacted more negatively to the anger vocalization and more positively to the joy vocalization. These results suggest that horses can match visual and vocal cues for the same emotion and can perceive the emotional valence of human non-verbal vocalizations.

**Abstract:**

Over the last few years, an increasing number of studies have aimed to gain more insight into the field of animal emotions. In particular, it is of interest to determine whether animals can cross-modally categorize the emotions of others. For domestic animals that share a close relationship with humans, we might wonder whether this cross-modal recognition of emotions extends to humans, as well. In this study, we tested whether horses could recognize human emotions and attribute the emotional valence of visual (facial expression) and vocal (non-verbal vocalization) stimuli to the same perceptual category. Two animated pictures of different facial expressions (anger and joy) were simultaneously presented to the horses, while a speaker played an emotional human non-verbal vocalization matching one of the two facial expressions. Horses looked at the picture that was incongruent with the vocalization more, probably because they were intrigued by the paradoxical combination. Moreover, horses reacted in accordance with the valence of the vocalization, both behaviorally and physiologically (heart rate). These results show that horses can cross-modally recognize human emotions and react emotionally to the emotional states of humans, assessed by non-verbal vocalizations.

## 1. Introduction

Over the last few years, an increasing number of studies have aimed to gain more insight into the field of animal emotions. Emotions can be expressed through different modalities: visually via body language and facial expressions [1], by vocal expressions [2], and by olfactory cues (e.g., Kikusui et al. [3]; Bredy & Barad [4]). The recognition of another’s emotions is a key aspect of sociality because this ability permits the correct reaction to these emotions (e.g., showing fearful submission to angry dominance), which facilitates social interactions and enhances group cohesion [5]. Several species have been shown to perceive the emotions of others (e.g., primates: [6,7,8,9]; dogs: [10,11,12]; sheep: [13]; and goats: [14,15]). However, it is still of question whether animals can categorize emotions. This ability can, for instance, be assessed by investigating the cross-modal recognition of emotions (i.e., integrating the information perceived by different senses [16]). In contrast to a simple discrimination between different emotions, which could be explained by simple associative rules, cross-modal recognition implies identifying the emotional messages expressed by the two modalities and interpreting these messages as being part of the same emotional category (e.g., joy vs, anger [10]). Cross-modal recognition of emotions enhances the efficiency, accuracy and reliability of the recognition [10,16]. Cross-modal recognition is usually assessed by a cross-modal preferential looking paradigm, in which two pictures are presented to the subject at the same time while a vocalization corresponding to one of the pictures is played. Subjects succeed by discriminating between the two pictures based on their congruency with the vocalization. For instance, dogs have been shown to match the size of a conspecific or the gender of a human experimenter with their voice [17,18,19]. To date, chimpanzees, rhesus monkeys, capuchin monkeys and dogs have been shown to cross-modally recognize conspecifics’ emotions using this paradigm [10,20,21,22]. 

Interestingly, dogs were shown to cross-modally recognize human emotions, as well [10]. Moreover, in a different paradigm they preferentially fetched an object towards which their owner had shown more positive emotions [23]. An interspecific recognition of emotions might indeed be highly beneficial for domestic animals, as they share their daily lives with humans and seem to have developed a high sensitivity to human behavior (e.g., Trösch et al. [24]; Ringhofer & Yamamoto [25]; Smith et al. [26]; Catala et al. [27]; Chijiiwa et al. [28]; Schuetz et al. [29]). Hence, we might wonder whether horses, another domestic species sharing a close relationship with humans, are also capable of cross-modally categorizing human emotions. It is particularly important to study how horses perceive and react to human emotions in the context of the horse-human relationship. In particular, if the valence of the emotions we express leads in turn to an emotional response in horses (e.g., a fear response for human negative emotions or on the contrary appeasement for positive emotions), it could impact horse welfare as well as the security of the users. The expression and processing of emotions have been well described in domestic horses (*Equus caballus*). They can communicate emotions both vocally [30] and visually (by body posture and facial features; e.g., Lansade et al. [31]). Horses can also discriminate emotions both intra- and interspecifically: they react differently when facing pictures of positive or negative facial expressions of both humans [26,32,33] and conspecifics [34] and when hearing positive or negative nonverbal vocalizations from humans [35] and conspecifics [30]. A recent study from Nakamura et al. [36] suggested that horses could recognize human emotions cross-modally in a violation of expectation paradigm (i.e., comparing their behavior when facing an expected and unexpected scene). A unique picture of an emotional facial expression (joy or anger) was projected on a white screen in front of the horses. The picture then disappeared, and horses heard a verbal expression (gentle or scolding). The horses looked at the white screen more quickly when hearing a vocalization that was incongruent with the emotional valence of the picture (and thus when there was a violation of their expectations) than when it was congruent. Seeing these promising results, Nakamura et al. [36] suggested to further investigate cross-modal recognition of human emotions in horses using the cross-modal preferential looking paradigm that had been used in dogs [10], for interspecific comparison purpose.

In this study, we investigated the cross-modal recognition of human emotions in horses using the cross-modal preferential looking paradigm. Horses were simultaneously shown two pictures of a human face (one expressing joy and the other expressing anger), while a non-verbal vocalization of either positive (joy) or negative (anger) valence was played by a speaker. Our aims were (1) to see whether the previous results [36] could be generalized with a different, widely used paradigm (the cross-modal preferential looking paradigm) and a large sample size; (2) to investigate whether horses react emotionally (behaviorally and physiologically) to other human non-verbal vocalizations than the ones used in the previous study [35]. Our hypotheses were that horses would discriminate between the two pictures (by looking preferentially at one of them) based on their congruency with the vocalization played and would react differentially as a function of the valence of the vocalizations.

## 2. Materials and Methods

### 2.1. Subjects and Husbandry

Thirty-four Welsh mares (*Equus caballus*, mean age ± SD = 9.47 ± 0.70) were used for this experiment. These horses were born at the French National Institute for Agricultural Research experimental unit (PAO, INRA, 2018. Animal Physiology Experimental Facility, DOI: 10.15454/1.5573896321728955E12) and were reared there for their whole life. During the experiment, the horses were kept inside in groups on straw bedding, with daily access to a paddock. They received straw, hay and water ad libitum. Although these horses are kept for research purpose and are not ridden, they are handled by humans on a daily basis and are very familiar with humans. None of them had participated in an experiment involving human emotions before.

### 2.2. Ethical Note

Our experiment received a positive recommendation from the Val de Loire Ethical Committee (CEEA VdL, Nouzilly, France). The animals were not food deprived during the experiment and did not undergo any invasive procedures. They lived in social groups and had daily access to an outside paddock.

### 2.3. Visual and Acoustic Stimuli

Horses were shown two animated pictures simultaneously, one on their left and one on their right, while a speaker (Ultimate Ears Megaboum 3, Newark, California, CA, USA) played a human non-verbal vocalization at 67 dB, 2 m away from the horse. The horses were tested with the same two animated pictures that came from the K-DEF database (F01-NE-HA and F01-NE-AN [37]). These pictures were validated by 168 people with 98% and 95% accuracy in identifying the emotion [37]. They consisted of videos (1 s) of a woman’s face either expressing joy or anger: the face was neutral in the beginning of the video and showed one of these emotions at the end (the timing of the emotional transition was identical for both animated pictures). The projected faces were approximately 2.5 times larger than a real person’s face. Moreover, we used two vocalizations that came from another validated database (M_6 and T_11 [38]). They were validated by 20 people with 100% and 95% accuracy in identifying the emotion [38]. They consisted of a woman’s voice pronouncing the (a) phonetic sound with different emotions. We chose vocalizations with the same duration as the animated pictures, one expressing joy and the other expressing anger.

### 2.4. Set-Up and Procedure

**Figure 1:**
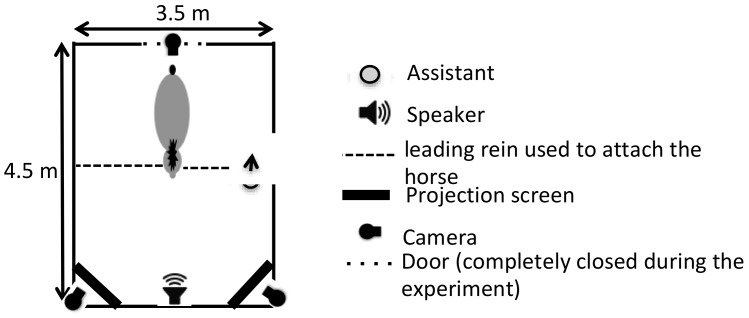
Schematic representation of the experimental set-up.

Horses were tested individually in a large familiar stall (3.5 × 4.5 m, Figure 1). They were attached by two leading reins (one on each side of the head) in front of two projection screens (1 × 2 m) and a speaker, which was placed between the screens. An assistant stayed with the horse during the test sessions (to ensure that the horse did not get entangled in the leading reins) but did not interact with the horse during the trials, keeping still and looking at the ground. Moreover, the assistant turned her back to the screens and was unaware of the side on which each animated picture was projected. Whether the assistant was standing on the left or on the right of the horse was counterbalanced between the horses.

The experiment took place in the following manner:

1. Familiarization. Horses were first familiarized with the experimental set-up by presenting two random pictures of nature while playing a recorded birdsong. The duration and number of sessions depended on the individual horse, but each horse was submitted to at least two familiarization sessions of a minimal duration of 5 min each. Familiarization stopped when the horse could stay attached in front of screen for 1 min without constraint with a heart rate lower than 80 bpm.
2. Test session. Horses were tested only once, always in the afternoon. A test session consisted of 6 trials (three with the “joy” vocalization and three with the “anger” vocalization) with 5 s breaks between trials (with black screens and no sound played). Each trial lasted 15 s: the same vocalization was repeated, and the animated pictures were played on a loop, one on the left and one on the right of the horse. Thus, for each vocalization, a horse had two trials with the matching picture on one side and one trial with the matching picture on the other side. The sides of the matching picture were counterbalanced between the horses so that at the group level, there were an equal number of trials with the matching picture on the right and on the left side. The order of the vocalizations and the side of each picture were semi-randomly distributed between the trials: the same vocalization and the same configuration of pictures were never presented more than twice in a row.

### 2.5. Coding and Statistical Analyses

During all sessions including familiarization, the horses were equipped with a heart monitor system (Polar Equine RS800CX Science, Polar Oy, Finland), and their behavior was filmed by 3 cameras (two at the front to code the horse behavior in response to the vocalizations and one at the back to code their looking duration towards the pictures). The following variables were later analyzed with BORIS software (v. 6.0.6 [39]), using the frame-by-frame mode (with 25 frames per second) for more precision. All the videos were analyzed by a same coder without sound, so that the coder was blind to the vocalization that was played. Moreover, a second coder analyzed 20% of the videos and the interobserver reliability was assessed for each behavioral measure by an Interclass Correlation Coefficient (ICC; [40]).

1. Preference index: this measure was calculated for each picture (congruent and incongruent with the vocalization played) to investigate the multimodality of the recognition of human emotions. This preference index was defined as the percentage of time spent looking at one picture across the total duration of the trial (15 s). Horses were considered to be looking at a picture when their muzzle was directed towards this picture (within 45° [25]). The ICC was 0.92 (lower bound = 0.89), which is considered as an excellent interobserver reliability [40].
2. Behavior in response to the valence of the vocalization: the percentage of time spent in a vigilant posture (the horse freezes, with the two ears oriented forwards and the head high [41,42,43]) and percentage of time spent in a relaxed posture (the horse relaxes its neckline muscles and puts its head down: angle between the neck and the withers is wider than 165°). For the vigilant posture, the ICC was 0.93 (lower bound = 0.87), which is considered as an excellent interobserver reliability [40]. For the relaxed posture, the ICC was 0.89 (lower bound = 0.80), considered as a good reliability [40].
3. Heart rate in response to the valence of the vocalization: the mean heart rate and the maximal value across the duration of the whole trial, and the difference in the mean heart rate between the first and last 5 s of the trial. As the trials followed each other closely and the heart rate might not have been reset to a basal level between the trials, we analyzed these variables during the first trial only. Due to technical issues with the heart monitor system, data were missing for several individuals (30 individuals were used to analyze the mean and maximal value of the heart rate, and 22 were used to analyze the difference in the heart rate between the first and last 5 s). The excluded individuals were average in their behavior (mean ± SEM of excluded individuals for the relaxed posture: 0.080 ± 0.023; global mean: 0.081 ± 0.008; mean of excluded individuals for the vigilant posture: 0.593 ± 0.075; global mean: 0.572 ± 0.020) and came from the two conditions (anger vocalization and joy vocalization). All horses were familiarized with the heart monitor system before the beginning of the experiment during the familiarization.

All statistical analyses were performed using R 3.0.2 (R Core Team, 2013). The significance threshold was fixed at 0.05. The preference index was analyzed with a generalized linear mixed model with a binomial distribution (as it is a percentage) using the “lmer” function in the lmerTest package [44]. The congruency and the valence of the picture were looked at, as well as the valence of the vocalization, and the interaction effect of the congruence of the picture by the valence of the vocalization were added as a fixed effect. The trial number nested in the identity of the horse was added as a random effect. The behavior of the horse in response to the vocalizations was analyzed with a generalized linear mixed model with a binomial distribution. The valence of the sound (joy or anger) was added as a fixed effect, and the identity of the horse was added as a random effect. The heart rate of the horse in response to the vocalizations was analyzed with a linear model, with the valence of the sound as a fixed effect. The residuals were checked graphically for normal distribution and homoscedasticity.

## 3. Results

**Figure 2:**
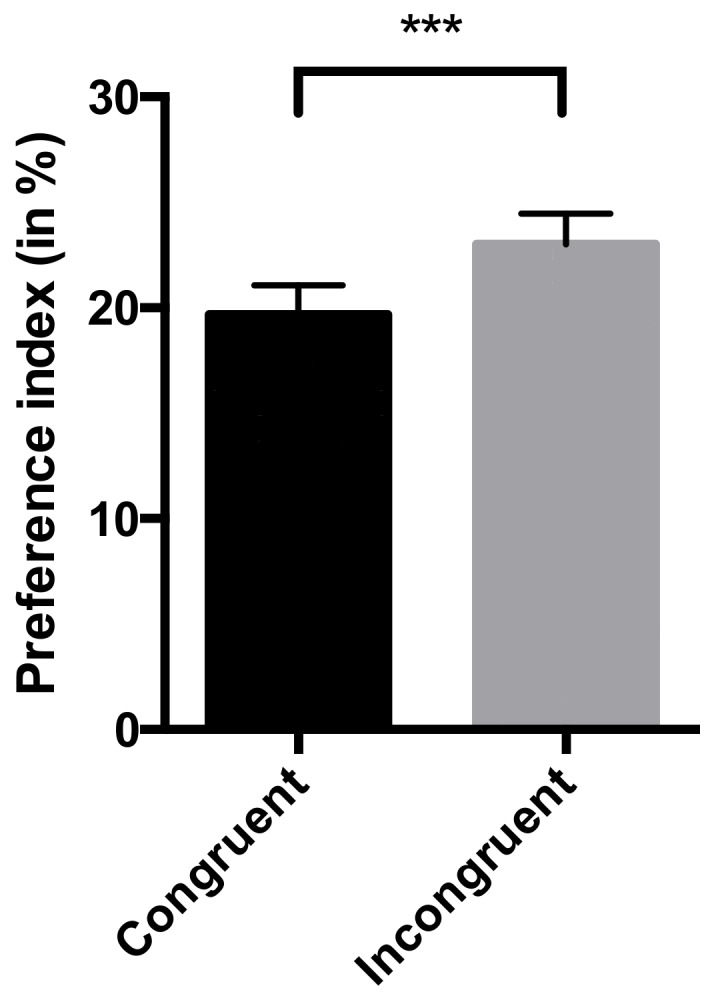
Effect of the congruence of each picture with the vocalization played on the preference index (i.e., the percentage of time spent looking at each picture) for that picture. Significance was assessed by a generalized linear mixed model: *** *p* < 0.001.

The preference index was significantly higher for pictures that were incongruent with the emotional valence of the vocalization played (X^2^ = 13.00, *p* < 0.001; Figure 2). This preference for the incongruent picture was not significantly affected by the valence of the vocalization (X^2^ = 3.28, *p* = 0.194). Besides the congruence of the picture, looking time was not significantly influenced either by the valence of the picture (X^2^ = 3.26, *p* = 0.071) or of the vocalization (X^2^ = 0.019, *p* = 0.890).

**Figure 3:**
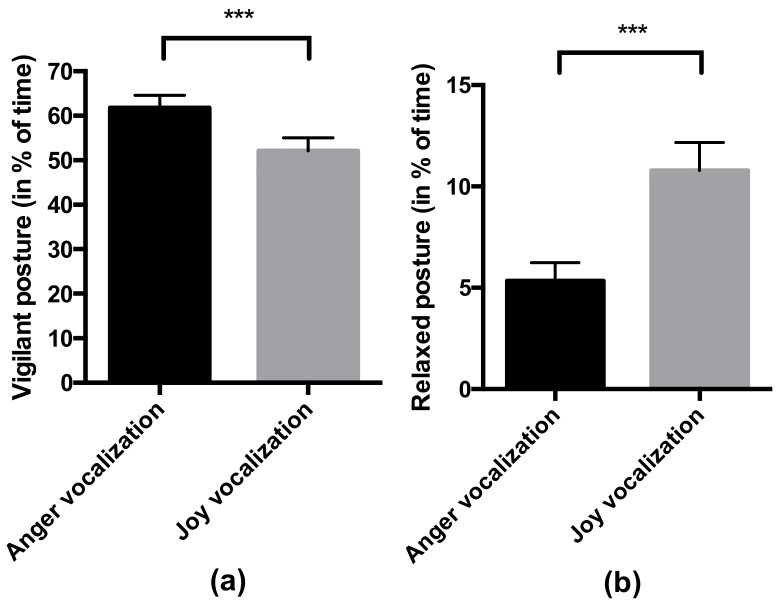
Effect of the emotional valence of the vocalization (joy or anger) on the behavior of the horse over the six trials: (**a**) Percentage of time spent in a vigilant posture; (**b**) Percentage of time spent in a relaxed posture. Significance was assessed by a generalized linear mixed model: *** *p* < 0.001.

Horses also behaved differently according to the valence of the vocalization. The percentage of time spent in a vigilant posture was significantly higher (F = 34.26, *p* < 0.001) during the anger vocalization than during the joy vocalization (Figure 3), while the percentage of time spent in a relaxed posture was significantly higher (F = 33.76, *p* < 0.001) during the joy vocalization than during the anger vocalization (Figure 3).

**Figure 4:**
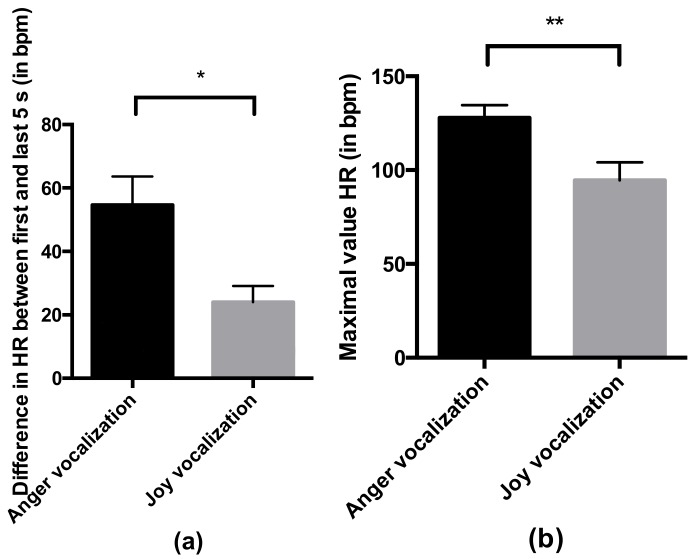
Effect of the emotional valence of the vocalization (joy or anger) on the heart rate of the horse during the first trial: (**a**) Difference in the mean heart rate between the first and last 5 s of the trial; (**b**) Maximal value of heart rate during the trial. Significance was assessed by a linear mixed model: * *p* ≤ 0.05, ** *p* < 0.01.

The difference in HR between the first and last 5 s of the trial (F = 6.83, *p* = 0.016) and the maximal HR value (F = 8.39, *p* = 0.007) were significantly higher during the anger vocalization than during the joy vocalization (Figure 4). The mean heart rate tended to be higher during the anger vocalization than during the joy vocalization (F = 3.83, *p* = 0.060).

## 4. Discussion

Our results show that the horses discriminated between the two pictures of human facial expressions based on their congruence with the non-verbal vocalization played during the presentation. Moreover, horses responded behaviorally and physiologically to the valence of human vocalizations.

### 4.1. Horses are Capable of Cross-Modal Recognition of Human Emotions

In line with our hypothesis, horses successfully discriminated between the two pictures of human faces (one expressing joy and the other expressing anger) based on their congruence with the vocalization they were hearing. These results suggest that horses, similar to dogs [10], are capable of interspecific multimodal recognition of human emotions in a cross-modal preferential looking paradigm. They could match the valence of the facial expression with the valence of the vocalization, meaning that they could assign these two stimuli that have different natures to the same correct emotional category.

Our study did not include any previous training, and since horses were tested with pictures and vocalizations of strangers, they could not have previously formed an association between the visual and vocal cues used in this experiment. Furthermore, as the pictures and the vocalizations came from different databases, there was no correlation between the timing of the facial movements observed on the animated pictures and the vocalization heard. Hence horses could not base their looking preference on that cue. However, as we only used one set of pictures and one set of vocalizations, further study would be necessary to test whether these results can be generalized to different pictures and vocalizations. Finally, it can be noticed that the percentages of time spent looking at each stimulus were relatively low (below 25%). This can be explained by the restrictive criterion we used to define this behavior (muzzle oriented within 45° towards the picture). Indeed, similarly low values are common in other studies using this criterion (e.g., Trösch et al. [24]; Ringhofer & Yamamoto [25]; Proops & McComb [45]; Lampe & Andre [46]).

Our results are consistent with and complete previous studies. First, it was shown that horses can discriminate human emotions based on their facial expression [26,32] or non-verbal vocalization [35]. These results are further supported by a recent study using the violation of expectations paradigm. In this study, they showed that horses looked at the screen more quickly when hearing a verbal vocalization that did not match the emotional valence of a previously seen human facial expression than when it was congruent [36]. Our results show that cross-modal recognition extends to non-verbal vocalizations using a different experimental paradigm.

Interestingly, horses looked preferentially at the incongruent picture, which is in contrast to the results found in primates and dogs in similar paradigm [10,17,18,19,20,21,22]. This result might be explained by differences in how these different species processed the task. Indeed, previous studies have shown that horses usually pay more attention to nonmatching stimuli than to matching stimuli. Indeed, in Nakamura et al. [36], horses responded more quickly and looked longer when the vocalization was incongruent with the facial expression than when the stimuli were congruent. Horses also looked more quickly, longer and more often when the voice they heard did not match the identity of a person they had previously seen [46,47]. An explanation for this result might be that horses paid more attention to the incongruent situation because they were intrigued by the paradoxical combination. When hearing the vocalization, they might have created an expectation to observe the corresponding facial expression. Seeing the incongruent picture might have violated these expectations and therefore engendered an increased interest compared to the expected congruent picture (similarly to the explanation given by Proops et al. [47] in a violation of expectation paradigm).

### 4.2. Horses Responded Behaviorally and Physiologically to the Valence of Non-Verbal Vocalizations

Horses showed significant physiological (heart rate) and behavioral differences when hearing joy and anger vocalizations. When hearing the joy vocalization, their heart rate was lower; they spent twice as much time in a relaxed posture and spent less time in a vigilant posture (even though they still expressed this behavior, probably because the test itself was unusual for them). The vigilant posture is considered to be a behavioral cue of negative emotional state in horses [41,42,43], while the relaxed posture is related to a positive emotional state [31]. Hence, it seems that the emotional reactions of the horses were consistent with the negative or positive valence of the vocalizations. Horses expressed more negative emotions during the anger vocalization and more positive emotions during the joy vocalization. Concerning the heart rate, data were missing for several individuals due to technical issues with the heart rate monitor. However, as the excluded individuals were average in their behavior and came from the two conditions (anger vocalization and joy vocalization), it is unlikely to have influenced our results. Hence, our results suggest that horses react emotionally to human emotions based on non-verbal vocalizations.

Horses are known to discriminate between whinnies of different valences from familiar horses (i.e., reunion and separation calls [30]). They also reacted more positively when hearing human laughter than human growling (in terms of behavior and left-hemisphere bias) [35]. Our study goes one step further as it shows that this ability extends to other types of vocalizations expressing joy and anger, as well. Moreover, we used two vocalizations consisting of the same phonetic (a) sound pronounced with either joy or anger. These vocalizations shared the same duration and acoustic characteristics (in contrast to growling and laughter, as laughter consists of voiced pulses separated by short pauses, by definition). Thus, the response of horses goes beyond simple acoustic differences between the vocalizations, strengthening the hypothesis that they reacted to human emotions.

## 5. Conclusions and Implications for the Horse-Human Relationship

Horses reacted differently, both in their physiology and behavior, when hearing vocalizations of joy and anger. More importantly, horses could associate sounds and pictures in the cross-modal recognition of human emotions, which suggests that horses are capable of categorizing emotion stimuli based on their valence, independent of their modality.

Understanding how horses perceive and react to human emotions is important as it has direct implications for horse management and welfare. Indeed, a human expressing negative emotions could cause stress for the horse and even lead to horse-related accidents because of a fear reaction. A human expressing positive emotions, on the contrary, could be appeasing for the horse and can be used in a training context.
